# Using Rasch analysis to assess the latent construct of the Capacity to Work Index in a Swedish working population sample

**DOI:** 10.1093/eurpub/ckaf001

**Published:** 2025-01-17

**Authors:** Agneta Blomberg, Gunnel Hensing, Monica Bertilsson, Emina Hadžibajramović

**Affiliations:** School of Public Health and Community Medicine, Institute of Medicine, Sahlgrenska Academy at the of University of Gothenburg, Gothenburg, Sweden; School of Public Health and Community Medicine, Institute of Medicine, Sahlgrenska Academy at the of University of Gothenburg, Gothenburg, Sweden; School of Public Health and Community Medicine, Institute of Medicine, Sahlgrenska Academy at the of University of Gothenburg, Gothenburg, Sweden; School of Public Health and Community Medicine, Institute of Medicine, Sahlgrenska Academy at the of University of Gothenburg, Gothenburg, Sweden; Institute of Stress Medicine, Region Västra Götaland, Gothenburg, Sweden

## Abstract

Measurements of capacity to work (CTW) in relation to common mental disorders (CMD) are needed to improve research on determinants for maintained work participation (WP). The aim of this study was to assess the construct validity of the Capacity to Work Index (C2WI) in a heterogenous sample of the Swedish working population. Cross-sectional web survey data among Swedish employees (*n* = 8201) was used. The construct validity was assessed in terms of the unidimensionality of the scale, response categories appropriateness, and the differential item functioning with respect to gender and age. Rasch analysis was used in both the full sample and randomly selected subsets. The Rasch model (RM) was fitted using two versions of the C2WI construct: the original 17-item scale and a 7-item scale. The 17-item scale did not discriminate as required, whereas the 7-item scale demonstrated a better fit to the RM. However, statistically significant χ^2^ statistics indicated non-invariant item performance across the latent continuum. A third attempt assessed both scales on a subgroup, yielding improved results, but an overall fit to the RM was not achieved. CTW in relation to CMD assessed by the C2WI did not fulfil the requirements for construct validity outlined by the RM. Real-world experiences of CTW are complex and reflect expressions of mental health in diverse work environments. Further studies are required to determine the predictive capacity of C2WI and its individual items in relation to relevant outcomes, such as maintained WP in the working population.

## Introduction

Common mental disorders (CMD) impact public health and work participation (WP) [1–7]. For preventive measures, early indicators of decreased WP are needed. A relevant focus is capacity to work (CTW) as a multidimensional phenomenon that balances CMD symptoms and work demands. However, specific measures are lacking [8]. A core argument for a specific instrument is the complex nature of CMD [9], and the variety of manifestations of CMD in work settings [10].

The generic Work Ability House model has four dimensions suggested to interact: health and functional capacities; competence; values, attitudes, motivation; and work, work community and management [11]. The Person-Environment-Occupational-model (PEOM) postulates a transactional and dynamic interplay between the individual and the occupational context [12]. Existing instruments for measuring work capacity include the Work Ability Index (WAI), Work Role Functioning, mini-ICF-APP, Lam Employment Absence and Productivity Scale, and Work Instability Scale-CMD are either generic or developed for clinical purposes [13–15]. A clinical population is characterized by symptoms or formal CMD diagnoses. The expected variation in CTW is lower in clinical populations compared with observations in the general working population, where a wide range of symptoms from subthreshold to formal diagnoses are present.

As a complementary CMD-specific approach, the Capacity to Work Index (C2WI) was developed to suit a working population and to contribute with details on how CTW varies. The C2WI was recently assessed in a field study [16]. The result indicated high internal reliability, and the principal component analysis (PCA) showed a loading on a single component when positively framed items were excluded. However, the field study had a small sample size and a homogenous work context, indicating limited generalizability. The aim of this study was to assess the construct validity of the C2WI in a heterogeneous sample of the Swedish working population. Specifically, the study focused on determining the unidimensionality of the C2WI as well as the appropriateness of response categories and the differential item functioning (DIF) with regards to gender and age.

## Methods

The ‘Work Participation and Mental Health at Work’ project is part of the network ‘New Ways’—‘mental health at work’ aiming for scientific knowledge to promote WP and prevent sickness absence. This cross-sectional study utilized data from a web-based survey of employed individuals aged 18 years and older.

### Recruitment process

There are no public registers of currently working employees in Sweden, so different recruitment procedures were used to obtain a large sample from heterogeneous work contexts. Initially, private sector employers and Swedish trade unions were contacted and provided with detailed information about the study. The recruitment of public sector employees was then conducted similarly. Employers who agreed to participate provided employees’ work emails. Study details, ethical considerations, and survey links were sent directly to the employees. Union members received the information from their union through newsletters via email. The initial data collection recruiting employees in the private sector yielded a limited number of participants (*n* = 397). Consequently, a web panel (the Norstat panel) was utilized, comprising individuals who are randomly contacted through telephone inquiries and consent to participate in the panel.

### Data collection

Data were collected during 24 November 2021 and 18 August 2022. The first data collection was conducted by the SOM Institute, a non-profit academic web-survey centre within the University of Gothenburg. The second data collection, utilizing the web panel, was conducted by an external survey company (Enkätfabriken, Gothenburg, Sweden). The survey included questions on sociodemographic information, employment sector, work characteristics, and health-related instruments. Survey links remained open for 4 weeks. Two reminders were sent to employees recruited through employers, whereas no reminders were sent to employees recruited through unions.

### Participants

The present study included participants with complete responses on gender (those identifying as non-binary were excluded due to small numbers, *n* = 35), age and C2WI, resulting in a study sample of *n* = 8201. A flowchart of the inclusion procedure and response rates are presented in Supplementary file 1, Fig. S1.

### Measures

#### The Capacity to Work Index

The C2WI consists of 17 statements (Table 2). The response options were: ‘Not at all’, ‘To a low degree’, ‘To a moderate degree’, ‘To a high degree’, and ‘I don’t know/it is not relevant’ (coded 1–5). Three positively framed statements were reversed (no. 4, 6, 10) before performing the Rasch analysis. The response option ‘I don’t know/it is not relevant’ was excluded since the intended increasing levels across the response categories did not apply. The frequency by which the respondents assessed ‘I don’t know/it is not relevant’ ranged between 0.9% (Item 3: ‘I have had difficulty prioritizing tasks’) and 6.7% (Item 8: ‘I have continued to work, even though it has caused mental or physical problems for me’).

#### WHO-5 Well-Being Index

The Swedish version of the World Health Organization—Five Well-Being Index (WHO-5) was utilized. The WHO-5 is a unidimensional index measuring overall subjective well-being over a 2-week period on a six-point Likert scale ranging from ‘All of the time’ (coded 5) to ‘Never’ (coded 0). As suggested by Bech (2012) [17], response options were summarized to form a scale from minimum 0 to maximum 100 points, with higher scores indicating higher level of mental well-being.

#### Age, education level, and work sector

Age was coded into three categories: 18–34, 35–54, and 55–74 years. Level of education was coded into three categories: university/higher education, upper secondary, and compulsory school. Work sector was measured with three categories: public sector, private sector, and ‘others’. The category ‘others’ was deemed too small (*n* = 117) and was excluded.

### Data analysis

Data were analysed using IBM SPSS Statistics Version: 29.0.0.0. Descriptive statistics are presented as frequencies and percentages. In the absence of established cut-off scores, the descriptive statistics for WHO-5 (Table 1) and C2WI (Table 2) are presented as follows: below or equal to the first quartile (Q1), between the first and the third quartile, and equal to or above the third quartile (Q3).

**Table 1.** Sociodemographic characteristics of the study population^a^ based on the Swedish ‘Work participation and mental health at work’ (ADAPT) research project, 2021–2022 (*n* = 8201^b^)

	*n* (%)
Gender	
Women	4769 (58)
Men	3432 (42)
Age groups^c^	
18–34 years	1908 (23)
35–54 years	4162 (51)
55–74 years	2131 (26)
Education level	
University/Higher education	3984 (49)
Upper secondary school	4057 (49)
Compulsory school	157 (2)
Work sector	
Public sector	4521 (56)
Private sector	3601 (44)
WHO-5 Well-being Index	
First quartile: ≤44	2200 (27)
Between first and third quartile: 45-76	4289 (53)
Third quartile: ≥77	1665 (20)
Long-term health conditions	
No long-term health conditions	3285 (40)
Yes, long-term mental health conditions	1276 (16)
Other long-term health conditions	3554 (44)

a The study population is not a simple random sample that might compromise representativity and generalizability.

b Dispersed numbers of participants are due to internal missing.

c Median = 46 years was used in the Rasch analysis (Q1 = 35, Q3 = 55).

**Table 2.** Descriptive statistics of the Capacity to Work Index (C2WI) in relation to CMD, based on the Swedish ‘Work participation and mental health at work’ (ADAPT) research project: 2021–2022, (*n* = 8201)

C2WI 17 item and C2WI 7 item version (shown in italic: no: 3, 8, 9, 10, 11, 14, 16)								
Response option:	Not at all (1)		To a low degree (2)		To a moderate degree (3)		To a high degree (4)	
The following statements refer to your job during the past week:	*n*	(%)	*n*	(%)	*n*	(%)	*n*	(%)
1. Disruptive noise prevented me from performing my job.	3400	(42)	2463	(30)	1675	(20)	663	(8)
2. Thinking has been tough and slow.	2363	(29)	2777	(34)	2155	(26)	906	(11)
*3. I have had difficulty prioritizing tasks*.	2939	(36)	2633	(32)	1867	(23)	762	(9)
4. I have been able to maintain the work pace required for my work.	4913	(60)	2052	(25)	640	(8)	596	(7)
5. I have had difficulty controlling my emotions.	4284	(52)	2140	(26)	1236	(15)	541	(7)
6. I have been sensitive to criticism from others.	3472	(42)	2595	(32)	1502	(18)	632	(8)
7. I have ‘put on a facade’ to allow me to be at work.	3952	(48)	1852	(22)	1367	(17)	1030	(13)
*8. I have continued to work even though it has caused mental or physical problems for me*.	4156	(51)	1528	(18)	1235	(15)	1282	(16)
*9. I have had to choose to not do free-time activities to have energy to work*.	3314	(40)	1837	(23)	1552	(19)	1498	(18)
*10. In the last week, I have gotten energy from and enjoyed my work task.*	913	(11)	1701	(21)	3467	(42)	2120	(26)
*11. have had difficulty learning new work tasks*.	4917	(60)	2148	(26)	899	(11)	237	(3)
12. I have felt like a stranger at work.	5692	(69)	1438	(18)	784	(10)	287	(3)
13. I have felt like I am closed off in ‘a bubble’, which has been a problem for me at work.	5637	(69)	1538	(18)	798	(10)	228	(3)
*14. I have avoided situations where many people physically or digitally meet because I do not have the energy to participate.*	4580	(56)	1692	(20)	1128	(14)	801	(10)
15. I have felt physically weak, sore, or tense, which has been an obstacle for me at work.	4507	(55)	2033	(25)	1141	(14)	520	(6)
*16. I have been able to stay calm.*	278	(3)	482	(6)	1861	(23)	5580	(68)
17. I have felt wound up.	1897	(23)	2757	(34)	2519	(31)	1028	(12)

The Rasch analysis was performed using RUMM2030 software [18], applying the partial credit model [19, 20]. The Rasch model (RM) is a psychometric model for analysing categorical data to evaluate whether the observed data satisfy the model assumptions. The RM is preferred over classical test theory as it does not require a normal score distribution [21]. Important concepts within the RM are unidimensionality (all items reflect a single latent construct, which is a requirement for combining items into a total score), monotonicity (item responses are positively related to the latent construct), invariance (items should work invariantly across the continuum of the construct for all individuals), DIF (given the same level of the latent construct items need to work invariantly for all comparable groups), and local dependency (having extracted the unidimensional latent trait, there should be no other meaningful patterns in the residuals).

The overall fit to the RM was assessed by a χ^2^ statistic (expected to be non-significant indicating invariance), and by means and standard deviations for the overall person and item fit residuals (expected values around 0 and 1 respectively), as well as by Andersen’s conditional likelihood ratio (CLR) [22] expected to be non-significant and calculated using the *iarm* package in R. Unidimensionality was investigated with the Smith’s test of unidimensionality [23]. To perform this test, first the PCA on the item residuals was conducted. Items loading positively and negatively on the first principal component were used to obtain an independent person estimate. Independent *t*-tests of mean differences calculated for person estimates of positively and negatively loaded items were performed for each participant. According to the Smith’s test, unidimensionality is indicated if <5% of these differences are found outside the range of ±1.96. The power of the C2WI scale to discriminate among respondents with different levels of CTW were assessed with the Person Separation Index (range 0–1) and presented only in case the good fit to the RM was achieved. Targeting of C2WI items and persons (on a logit scale) was evaluated using a person-item-threshold graph comparing the distribution of person estimates with item thresholds. Multiple individual item fit indicators were investigated: threshold ordering (appropriateness of response categories implying sequentially ordered thresholds across the latent trait for each item), item fit residuals (expected to be in the interval of ±2.5 with a non-significant χ^2^ statistic, item’s ability to discriminate), pairwise residual correlations (expected to be <0.2 above the average correlation [24], local independence), and DIF for age and gender analysed by ANOVA on standardized item residuals. Significance level was set to 0.01 and Bonferroni adjusted in DIF analyses. Item fit was assessed graphically by means of the item characteristic curves and by threshold probability curves. DIF was analysed for age (under or above median age of 46) and gender (women and men). Person fit was evaluated by means of person residuals, and the percentage of participants with high residual values was reported (outside the interval of ±2.5). Given the large dataset, even minor differences can attain statistical significance. To address this, the evaluation of the fit to the RM was conducted on samples of varying sizes (*n* = 500, *n* = 800, *n* = 1000). The random samples were drawn out of the complete cases using the select random cases function in SPSS. To enhance the robustness of the results, two random datasets were drawn for each sample size: *n*  = 1000, *n* = 800, and one dataset for *n* = 500. This approach permits a comprehensive understanding of the RM fit, thus mitigating the potential data fluctuations.

#### A comparative analysis of two versions of the C2WI

We fitted the RM to two versions of the C2WI; the 17-item version as developed by Hensing et al. (2024) [16] and a 7-item version guided by theoretical reasoning [25] based on the PEOM, as described by Law et al. [12]. The reduction in items was also guided by an ambition to exclude items that could be interpreted as too closely related to symptoms. Researchers A.B., E.H., M.B., and G.H. individually assessed each of the 17 items from the two theoretical perspectives. After individual assessments, the item reduction was discussed. Disagreements were identified and resolved. Eight items (no. 2, 5, 6, 7, 12, 13, 15, and 17) were categorized as person-related, and two items (no. 1 and 4) as occupational factors. Seven items (no. 3, 8, 9, 10, 11, 14, and 16) were categorized as environmental factors and thus selected for additional testing.

#### Subgroup analysis

To determine if individuals with mental health problems were more prone to grasp the essence of the C2WI, a subgroup analysis was conducted. The subgroup consisted of participants who scored at or below the first quartile of the WHO-5 index (≤44), and by selecting the option ‘mental health problem’ when responding to the question: ‘Do you have a chronic illness, problem or disability’ (*n* = 1276).

#### Ethical considerations

The project received ethical approval by the Regional Ethical Review Board in Gothenburg (no. 783-106) and by The Swedish Ethical Review Authority (no. 2020-06031). The introductory letter informed employees that the study was independent of the employer, voluntary, and that they could withdraw at any time. It also provided details of their legal rights under the General Data Protection Regulation.

## Results

### Descriptives

As can be seen in Table 1, the median WHO-5 score was 64 (Q1 ≤ 44, Q3 ≥ 77), and 16% reported having a long-term mental health condition. Self-assessments of the C2WI are presented in Table 2. The response category ‘Not at all’ (1) was used most frequently except for Item 2: ‘Thinking has been tough and slow’, and Item 17: ‘I have felt wound up’, with a more even distribution. Item 9: ‘I have had to choose to not do free-time activities to have energy to work’ displayed an even higher proportion of equal distribution between the response categories.

### Overall model fit of the C2WI

Overall model fit statistics for all random datasets and the total sample are presented in Table 3. Results indicated poor fit according to item and person residual mean and SD values. The χ^2^ statistic and CLR test were high and significant, indicating that the hierarchical ordering of the items was not invariant across the latent dimension of the work capacity continuum. The Smith’ test indicated problems with the dimensionality of the scale, with the percentage of misfitting pairs ranging between 6.4% and 15.8% and lower confidence bound being >5% in all datasets.

**Table 3.** Overall model fit of the C2WI, all samples, Andersen’s conditional likelihood (CLR) test, degrees of freedom (DF)

	Item residual		Person residual		Chi square		CLR			Unidimensionality
Datafile	Mean	SD	Mean	SD	Value	*P*-value		Value (DF)	*P*-value	Test % (95% CI)
Analysis with 17 items										
(1) *n* = 1000	−1.04	6.14	−0.21	1.16	2031.10	<.0001	1097	50	<.0001	6.4 (5.1; 8.2)
(2) *n* = 1000	−0.85	6.94	−0.20	1.21	2362.76	<.0001	1371	50	<.0001	15.8 (13.6; 18.2)
(3) *n* = 800	−0.75	6.37	−0.17	1.25	2165.43	<.0001	1086	50	<.0001	8.7 (6.9; 10.9)
(4) *n* = 800	−0.69	6.16	−0.19	1.18	1905.96	<.0001	970	50	<.0001	13.4 (11.3; 16.0)
(5) *n* = 500	−0.36	5.15	−0.16	1.23	1173.65	<.0001	581	50	<.0001	15.7 (12.7; 19.1)
(6) *n* = 8201	−2.76	18.59	−0.21	1.18	17461.31	<.0001	9745	50	<.0001	7.4 (6.9; 8.0)
Analysis with seven items										
(1) *n* = 1000	−0.76	3.43	−0.38	0.94	240.44	<.0001	136	20	<.0001	1.4 (0.08; 2.4)
(2) *n* = 1000	−0.51	3.66	−0.29	0.96	335.45	<.0001	127	20	<.0001	2.6 (1.7; 3.8)
(3) *n* = 800	−0.45	3.17	−0.29	0.98	226.24	<.0001	125	20	<.0001	2.1 (1.3; 3.4)
(4) *n* = 800	−0.64	3.08	−0.29	0.96	266.29	<.0001	143	20	<.0001	1.6 (0.09; 2.8)
(5) *n* = 500	−0.13	2.34	−0.25	0.97	143.25	<.0001	107	20	<.0001	5.3 (3.6; 7.7)
(6) *n* = 8201	−1.83	10.10	−0.29	0.95	1975.38	<.0001	1119	20	<.0001	1.1 (0.09; 1.3)
Subgroup analysis with seven items										
(7) *n* = 1000	0.45	2.49	−0.20	0.96	164.50	<.0001	89.1	20	<.0001	3.0 (2.1; 4.3)
(8) *n* = 800	0.26	2.53	−0.22	0.99	188.93	<.0001	125	20	<.0001	3.2 (2.2; 4.7)
(9) *n* = 500	0.14	2.02	−0.20	0.96	109.96	<.0001	58.6	20	<.0001	4.4 (2.9; 6.6)

### Rasch analysis on the two versions of the C2WI

Individual item fit was assessed using multiple sample sizes as indicators, and items showing misfit are summarized in Table 4. Looking at the first random sample (*n* = 1000) as an example, 14 out of 17 items showed significant misfit according to the item fit residuals, which were outside the predefined range of ±2.5 and statistically significant (for complete tables of item fit statistics, see Supplementary files 2 and 3). The most serious misfit was found for the reversed items (no. 4, 10, 16) for which item fit residuals were positive, indicating underdiscrimination. The results were confirmed by visual inspection (Supplementary file 4, Fig. S2), showing item characteristic curve for Item 4 as an illustrative example. The solid line shows the distribution according to adequate item fit and the dots are observed values. Same items are also identified as problematic regarding DIF for age and/or gender and threshold ordering (complete tables of variance of residuals DIF in all samples, see Supplementary file 5). Pairwise correlations between items indicated that several pairs of items had higher values than expected under the condition of local independence (Table 4 and Supplementary files 6 and 7).

**Table 4.** Items showing misfit according to various items fit statistics

Datafile	Item residual*	Residual correlations	DIFage	DIFgender	Disorder thresholds
Analyses with 17 items					
(1) *n* = 1000	**2, 4–16**	2**–**3, 5**–**6, 7**–**8, 8**–**9, 8**–**15, 12**–**13	4, 8	4, 8, 16	4, 8, 16
(2) *n* = 1000	**2–10**, 12, **13–17**	2**–**3, 5**–**6, 7**–**8, 8**–**9, 10**–**16, 12**–**13	2, 4, 7, 12	2, 4, 6, 9, 10	4, 16
(3) *n* = 800	**2, 4, 5, 6–10, 12–16**	2**–**3, 5**–**6, 8**–**9, 12**–**13	4, 7, 11	4, 7, 9	4, 9, 13, 16
(4) *n* = 800	**2, 4–10, 12–16**	2**–**3, 5**–**6, 8**–**9, 8**–**15, 12**–**13	2, 7, 11	4, 7	4, 8, 16
(5) *n* = 500	**2, 4, 5, 7, 9, 10, 13, 15, 16**	2**–**3. 2**–**11, 5**–**6, 8**–**9, 10**–**16, 12**–**13	7	4	4, 7, 10, 12, 16
(6) *n* = 8201	**1–16**	2**–**3, 5**–**6, 8**–**9, 8**–**15, 12**–**13	2, 4, 5, 7, 10, 11, 16	1**–**4, 6**–**10, 12, 13, 15	4, 8, 12, 16
Analyses with seven items					
(1) *n* = 1000	**8, 9, 10, 14**	8**–**9, 3**–**11	**–**	8, 9, 10	8, 16
(2) *n* = 1000	**8, 9, 10, 14**	8**–**9, 3**–**11	**–**	9, 10	16
(3) *n* = 800	**8, 9, 10, 14, 16**	8**–**9	11	9	16
(4) *n* = 800	**8, 9, 10, 16**	8**–**9	11	8	8, 14, 16
(5) *n* = 500	**8, 9,** 10**, 16**	8**–**9, 3**–**11	**–**	9	10, 16
(6) *n* = 8201	**8, 9, 10, 14, 16**				8, 16
Sub-group analyses with seven items					
(7) *n* = 1000	**8, 9, 10**	8**–**9, 3**–**11	**–**	9, 10	**–**
(8) *n* = 800	**8, 10**		11	9, 10	**–**
(9) *n* = 500	**8**, 10, **14**	8**–**9	**–**	**–**	8, 9

* Bold indicates statistically significant misfit.

The initial assessment of the 17-item scale indicated that the items did not discriminate as required, and the overall fit to the RM was poor. As a next step, the Rasch analysis was performed on the seven-item scale. The results for the overall model fit and the item fit indicators are summarized in Tables 3 and 4, respectively. Although, the Smith’s test indicated unidimensionality, the overall fit to the RM remained problematic, as evidenced by the high and statistically significant values of the χ^2^ statistic and CLR test in all analyses. Figure S3 (Supplementary file 8) shows the distribution of item thresholds and participants along the common logit scale (higher scores indicate a stronger reduction of CTW). A group of participants with very low locations (below −2 logits) falls below the range measured by the items. This is reflected by the person mean of −0.808 (SD 0.747) compared to the item mean (constrained to 0) and confirmed by the item frequency distributions. The highest response category is rarely used with most responses in the first two categories (Table 2), indicating suboptimal targeting. A similar pattern was observed for the seven-item version, but with more gaps as expected due to fewer number of items and across all random samples. The percentage of participants with high residuals ranged from 4.6% to 7.8% in the 17-item analyses and from 7.2% to 8.6% in the 7-item analyses across random samples.

### Rasch analysis using a subgroup

A subgroup analysis was done by testing the seven-item scale among participants reporting low mental well-being in random samples of varying sizes (*n* = 1000, *n* = 800, and *n* = 500). The overall fit to the RM was not achieved (Table 3, analysis 7–9). The response options worked better for the subgroup as fewer disordered thresholds were observed. Targeting was better, and persons and item thresholds better aligned, but again with gaps (see Supplementary file 8, Fig. S3 for illustrative example). Percentage of participants with misfitting residuals ranged between 1.7% and 2.1%.

## Discussion

The objective of this study was to determine the construct validity of the C2WI using the RM. In particular, the study sought to ascertain the unidimensionality of the instrument, the appropriateness of the response categories, and the presence of DIF with regard to gender and age. The 17-item scale did not reach predetermined levels of fit in any of the analyses. The seven-item scale indicated unidimensionality; yet, the overall fit to the RM remained deficient, and multiple indicators on the item level showed a lack of fit. The result for the subgroup reporting low mental well-being showed a better fit to the RM, with fewer items exhibiting disordered thresholds and better targeting compared to the corresponding analysis in previous tests. However, this was not sufficient to distinguish between response categories.

The result indicates multidimensionality, which is consistent with the theoretical understanding of CTW as a phenomenon that occurs in a dynamic interaction between the individual and the work context. CTW is a multifaceted construct that encompasses a range of elements, including knowledge, skills, abilities and motivation. These elements are shaped by the specific social settings in which they are practised. CTW is best understood as a continuum, from excellent to very poor; thus, the latent construct is not absolute; rather, it is relative to the context in which human performance occurs.

The C2WI was developed to advance the understanding of how CTW is reduced. The WAI contains open-ended questions such as ‘current work ability compared with the lifetime best’ and ‘work ability in relation to the demands of the job’. Such questions reflect ‘that’ the work ability is reduced, and to what extent, but they do not really provide information about ‘how’. The C2WI was developed in a multiple step process [26], based on qualitative analysis of personal experiences [16]. In constructing the instrument, Hensing et al. (2024) [16] incorporated three aspects of the PEOM to provide an instrument reflecting the complexity of CTW. The ambition to reflect multiple aspects may have counteracted unidimensionality. Nevertheless, the subgroup analysis of participants reporting low mental well-being demonstrated a stronger tendency towards measuring a latent construct with better targeting and lower percentage of participants with misfitting residuals. This may be indicative of the fact that the qualitative studies from which the instrument was developed comprised individuals with experience of CMD. This may have introduced a possible bias toward more severe expressions of reduced CTW. In a general working population, the range of experiences is expected to be broad, and statements need to cover this variation. C2WI should provide the ability to mirror experiences of WP with full CTW to a very reduced CTW.

In future studies comparing C2WI with other instruments measuring relevant aspects for WP, such as competence and work-related self-efficacy, could give complementary information to the construct validity, such as content validity and possible correlations between the different constructs. While the C2WI did not meet the Rasch criteria for construct validity, it may nonetheless reflect a satisfactory content validity and prove useful in predicting WP.

The study population represents different sectors, occupations, gender, age groups and varies in several other ways. We consider this, as well as the large sample size, a strength, as multiple experiences and work contexts contribute to the analysis. The sample size may have been unnecessary large, but since data were not collected for the Rasch analysis only, we decided to use the full sample. Danielsson et al. (2020) [14] concluded that their sample was too small and recommended a larger sample size.

In our study, we used multiple techniques to overcome the risk with χ^2^ statistic bias in large sample sizes. We used several randomly selected sub-samples (*n* = 1000, 800, 500), and rather than utilizing the conventional 0.05 significance level in the overall test, a conservative approach was employed (threshold reduced to 0.01). As item fit value also is susceptible to large sample sizes, Bonferroni-adjusted *P*-values were used. Additionally, a CLR-test of Andersen was performed [22].

Both the full version and a short version of the instrument were validated. It has been suggested in the literature that determining which items to include in an instrument should be guided by considerations other than just psychometric properties [27]. Prior to conducting subsequent data analysis, both *a priori* item reduction based on theoretical considerations and evaluations by experts were performed to define items for a short version (seven items). Moreover, data based on a sub-group analysis proved to better fit the RM. The knowledge gained from this analysis will be an asset in the future development of the C2WI.

Available softwares for doing Rasch analyses have pros and cons. We used the RUMM2030 in which, as mentioned earlier, the global test of model fit is sensitive to larger sample sizes. Measures described in the previous paragraph contributed to overcome some of these problems.

## Conclusion

CTW in relation to CMD assessed by the C2WI did not meet the criteria for construct validity as outlined by the RM. The findings can serve to reinforce the understanding of the CTW-construct as multidimensional. Real-world experiences of CTW are multifaceted and reflect expressions of mental health in a variety of work settings. The results were discouraging but the attempt is justified given the lack of instruments determining CTW as a factor that may maintain WP in relation to CMD in a working population. When C2WI was assessed in a subgroup comprising participants with low mental well-being, the instrument demonstrated a stronger tendency towards attaining the predetermined level of fit to the RM.

## Supplementary Material

ckaf001_Supplementary_Data

## Data Availability

The data are not publicly available due to containing information that could compromise the privacy of the study participants. Reasonable inquiries about access may be sent to School of Public Health and Community Medicine, Institute of Medicine, Sahlgrenska Academy, University of Gothenburg, P.O Box 453, SE-405 30 Gothenburg, Sweden, or by contacting: dataskydd@gu.se. The Swedish Ethical Review Authority will then be contacted for permission. Key points This study assessed the latent construct of the C2WI in a heterogenous sample of the working population in Sweden, based on cross-sectional data. Although two versions of the C2WI constructs were assessed, the results showed an overall poor fit to the Rasch model. When the C2WI was assessed in a subgroup based on participants with mental health problems, the model fit slightly improved. Further studies are required to determine the predictive capacity of C2WI in relation to relevant outcomes, such as maintained work participation in the working population.
