# Supplementary material for: Using Rasch analysis to assess the latent construct of the Capacity to Work Index in a Swedish working population sample
Source: Eur J Public Health. 2025 Jan 17;35(3):528–33. doi: 10.1093/eurpub/ckaf001 (PMC12187450; doi:10.1093/eurpub/ckaf001)
Supplement: ckaf001_Supplementary_Data [file ckaf001_supplementary_data.zip › ckaf001_Supplementary_Data/ejph-2024-06-om-0393-File007.docx]

Supplementary file 2.

Table 1. Fit Residuals, location, and threshold values from the C2WI17 analysis containing 17 items, subsample 1. n=1000.

|  |  |  | **Thresholds** | | |
| --- | --- | --- | --- | --- | --- |
| **Item** | **Fit Residuals*** | **Location** | **1*** | **2*** | **3*** |
| C2WI1 | **2.3** | -0.01667 | -0.77498 | -0.02006 | 0.745012 |
| C2WI2 | **-4.631** | -0.43141 | -1.52088 | -0.48829 | 0.714944 |
| C2WI3 | -1.069 | -0.17794 | -1.21411 | -0.11819 | 0.798495 |
| C2WI4 | **16.781** | -1.33691 | **-1.08526** | **-1.51997** | **-1.40551** |
| C2WI5 | **-3.531** | 0.1952 | -0.54168 | 0.351023 | 0.776256 |
| C2WI6 | **-3.277** | 0.08593 | -1.00011 | 0.121785 | 1.136119 |
| C2WI7 | **-7.303** | -0.12837 | -0.47369 | -0.08779 | 0.17637 |
| C2WI8 | **-6.425** | -0.19375 | **-0.20142** | **-0.33039** | **-0.04945** |
| C2WI9 | -3.032 | -0.36967 | -0.59771 | -0.38653 | -0.12479 |
| C2WI10 | **6.731** | -0.39617 | -1.56782 | 0.064319 | 0.314996 |
| C2WI11 | -2.872 | 0.629722 | -0.24725 | 0.756715 | 1.379705 |
| C2WI12 | -3.281 | 0.699393 | 0.526633 | 0.770106 | 0.801439 |
| C2WI13 | **-6.682** | 0.981421 | 0.412187 | 0.589981 | 1.942093 |
| C2WI14 | **-4.114** | 0.14052 | -0.13384 | 0.092284 | 0.463114 |
| C2WI15 | **-4.27** | 0.26838 | -0.16894 | 0.148199 | 0.825877 |
| C2WI16 | **6.376** | 0.59403 | **0.345203** | **0.895379** | **0.541508** |
| C2WI17 | 0.613 | -0.5437 | -1.67469 | -0.68039 | 0.723969 |

*Bold indicates statistically significant misfit

Table 2. Fit Residuals, location, and threshold values from the C2WI17 analysis containing 17 items, subsample 2. n=1000.

|  |  |  | **Thresholds** | | |
| --- | --- | --- | --- | --- | --- |
| **Item** | **Fit Residuals*** | **Location** | **1*** | **2*** | **3*** |
| C2WI1 | 2.336 | -0.05371 | -0.75087 | -0.08348 | 0.673229 |
| C2WI2 | **-5.435** | -0.35987 | -1.60123 | -0.45492 | 0.97653 |
| C2WI3 | **-2.022** | -0.201 | -1.3747 | -0.12438 | 0.896067 |
| C2WI4 | **18.2** | -1.3616 | **-1.3635** | **-1.2689** | **-1.4524** |
| C2WI5 | **-5.892** | 0.337533 | -0.40866 | 0.372599 | 1.048665 |
| C2WI6 | **-2.721** | 0.003224 | -0.913 | 0.151691 | 0.770984 |
| C2WI7 | **-7.056** | -0.06487 | -0.45734 | -0.17402 | 0.436744 |
| C2WI8 | **-3.48** | -0.11728 | -0.21923 | -0.19304 | 0.060424 |
| C2WI9 | **-5.749** | -0.42084 | -0.71779 | -0.45811 | -0.08662 |
| C2WI10 | **10.748** | -0.4728 | -1.67369 | -0.08119 | 0.336479 |
| C2WI11 | -1.619 | 0.587944 | -0.25442 | 0.517884 | 1.500372 |
| C2WI12 | -3.569 | 0.805077 | 0.30487 | 0.516608 | 1.593752 |
| C2WI13 | **-6.593** | 0.882421 | 0.405846 | 0.490959 | 1.750458 |
| C2WI14 | **-4.96** | 0.069628 | -0.12985 | -0.0835 | 0.422233 |
| C2WI15 | **-3.762** | 0.324838 | -0.36126 | 0.376219 | 0.959553 |
| C2WI16 | **7.867** | 0.611665 | **0.38126** | **1.091917** | **0.361817** |
| C2WI17 | -0.718 | -0.57036 | -1.84235 | -0.76584 | 0.897112 |

*Bold indicates statistically significant misfit

Table 3. Fit Residuals, location, and threshold values from the C2WI17 analysis containing 17 items, subsample 3. n=800.

|  |  |  | **Thresholds** | | |
| --- | --- | --- | --- | --- | --- |
| **Item** | **Fit Residuals*** | **Location** | **1*** | **2*** | **3*** |
| C2WI1 | **1.907** | -0.0487 | -0.8161 | 0.020882 | 0.649109 |
| C2WI2 | **-4.734** | -0.38565 | -1.56055 | -0.42431 | 0.827911 |
| C2WI3 | -1.628 | -0.10926 | -1.12786 | -0.14563 | 0.94573 |
| C2WI4 | **19.238** | -1.20405 | **-1.0128** | **-1.16692** | **-1.43244** |
| C2WI5 | **-4.078** | 0.296817 | -0.40959 | 0.118209 | 1.181826 |
| C2WI6 | **-2.371** | 0.091374 | -0.9255 | -0.05141 | 1.251029 |
| C2WI7 | **-6.897** | -0.13005 | -0.47973 | -0.1107 | 0.200272 |
| C2WI8 | **-4.916** | -0.16626 | -0.23186 | -0.15663 | -0.11028 |
| C2WI9 | -3.533 | -0.47134 | **-0.79242** | **-0.30838** | **-0.31321** |
| C2WI10 | **6.582** | -0.38048 | -1.53945 | -0.08041 | 0.478439 |
| C2WI11 | -0.95 | 0.618015 | -0.13665 | 0.607732 | 1.382959 |
| C2WI12 | -2.866 | 0.612546 | 0.266971 | 0.619185 | 0.951481 |
| C2WI13 | **-5.13** | 0.840513 | **0.442975** | **0.374875** | **1.70369** |
| C2WI14 | **-3.425** | 0.102741 | -0.18323 | 0.048722 | 0.442727 |
| C2WI15 | **-5.527** | 0.254509 | -0.41084 | 0.335959 | 0.83841 |
| C2WI16 | **5.593** | 0.63275 | **0.313636** | **1.065327** | **0.519287** |
| C2WI17 | -0.05 | -0.55349 | -1.84782 | -0.61361 | 0.800973 |

*Bold indicates statistically significant misfit

Table 4. Fit Residuals, location, and threshold values from the C2WI17 analysis containing 17 items, subsample 4. n=800.

|  |  | |  | | | **Thresholds** | |
| --- | --- | --- | --- | --- | --- | --- | --- |
| **Item** | **Fit Residuals*** | **Location** | | | **1*** | **2*** | **3*** |
| C2WI1 | -0.033 | -0.39031 | | -1.64601 | | -0.31663 | 0.791719 |
| C2WI2 | **-3.914** | -0.17831 | | -1.25953 | | -0.12536 | 0.84994 |
| C2WI3 | **-1.365** | -1.33133 | | **-1.28798** | | **-1.27654** | **-1.42947** |
| C2WI4 | **18.805** | 0.319381 | | -0.47793 | | 0.351673 | 1.084395 |
| C2WI5 | **-3.739** | 0.03849 | | -0.85459 | | 0.061408 | 0.908654 |
| C2WI6 | -2.658 | -0.03454 | | -0.38349 | | -0.07698 | 0.356849 |
| C2WI7 | **-6.793** | -0.20732 | | **-0.05865** | | **-0.30584** | **-0.25747** |
| C2WI8 | -2.988 | -0.42458 | | -0.74415 | | -0.59806 | 0.068486 |
| C2WI9 | **-4.811** | -0.439 | | -1.67038 | | 0.049043 | 0.304332 |
| C2WI10 | **7.324** | 0.627279 | | -0.0806 | | 0.756727 | 1.205713 |
| C2WI11 | -0.722 | 0.846729 | | 0.424546 | | 0.506697 | 1.608944 |
| C2WI12 | -3.051 | 0.88119 | | 0.346851 | | 0.814666 | 1.482054 |
| C2WI13 | **-5.627** | 0.088125 | | -0.03327 | | 0.023266 | 0.274378 |
| C2WI14 | -2.979 | 0.212776 | | -0.30429 | | 0.160679 | 0.781939 |
| C2WI15 | **-3.055** | 0.638996 | | **0.34175** | | **1.027867** | **0.547372** |
| C2WI16 | **5.358** | -0.56825 | | -1.83816 | | -0.57839 | 0.71181 |
| C2WI17 | -0.033 | -0.39031 | | -1.64601 | | -0.31663 | 0.791719 |

*Bold indicates statistically significant misfit

Table 5. Fit Residuals, location, and threshold values from the C2WI17 analysis containing 17 items, subsample 5. n=500.

|  |  |  | **Thresholds** | | |
| --- | --- | --- | --- | --- | --- |
| **Item** | **Fit Residuals*** | **Location** | **1*** | **2*** | **3*** |
| C2WI1 | -0.186 | -0.05841 | -0.80549 | -0.13685 | 0.767106 |
| C2WI2 | **-3.021** | -0.43137 | -1.67894 | -0.4736 | 0.858425 |
| C2WI3 | -1.741 | -0.15465 | -1.16028 | -0.07132 | 0.76764 |
| C2WI4 | **15.15** | -1.2663 | **-0.94675** | **-1.34386** | **-1.5083** |
| C2WI5 | **-3.189** | 0.294085 | -0.51913 | 0.238093 | 1.163289 |
| C2WI6 | **-2.364** | -0.04935 | -0.95765 | -0.10259 | 0.912175 |
| C2WI7 | **-4.516** | -0.12394 | **-0.55582** | **0.202326** | **-0.01832** |
| C2WI8 | -1.727 | -0.21048 | -0.33035 | -0.2534 | -0.04769 |
| C2WI9 | **-3.833** | -0.42885 | -0.87054 | -0.38547 | -0.03055 |
| C2WI10 | **6.328** | -0.55216 | **-1.72333** | **0.102465** | **-0.0356** |
| C2WI11 | -1.358 | 0.936354 | -0.18469 | 0.765498 | 2.228249 |
| C2WI12 | -1.657 | 0.830237 | **0.657256** | **0.399846** | **1.43361** |
| C2WI13 | **-4.794** | 0.93153 | 0.37852 | 0.514035 | 1.902035 |
| C2WI14 | -2.3 | 0.067921 | -0.25093 | 0.191269 | 0.263429 |
| C2WI15 | -2.923 | 0.365463 | -0.29778 | 0.148204 | 1.245965 |
| C2WI16 | **6.987** | 0.426348 | **0.274376** | **1.028737** | **-0.02407** |
| C2WI17 | -0.898 | -0.57643 | -1.72055 | -0.60817 | 0.59944 |

*Bold indicates statistically significant misfit

Table 6. Fit Residuals, location, and threshold values from the C2WI17 analysis containing 17 items, subsample 6. N=8201.

|  |  |  | | **Thresholds** | | |
| --- | --- | --- | --- | --- | --- | --- |
| **Item** | **Fit Residuals*** | **Location** | **1*** | | **2*** | **3*** |
| C2WI1 | **5.651** | -0.03174 | -0.75983 | | -0.14811 | 0.812711 |
| C2WI2 | **-13.721** | -0.41108 | -1.62747 | | -0.41579 | 0.810019 |
| C2WI3 | **-3.818** | -0.18434 | -1.13825 | | -0.23033 | 0.815549 |
| C2WI4 | **51.599** | -1.33335 | **-1.15378** | | **-1.46374** | **-1.38255** |
| C2WI5 | **-11.955** | 0.249574 | -0.42803 | | 0.219503 | 0.957253 |
| C2WI6 | **-9.185** | 0.029054 | -0.91102 | | 0.068942 | 0.929244 |
| C2WI7 | **-20.341** | -0.1068 | -0.48976 | | -0.17501 | 0.344362 |
| C2WI8 | **-15.125** | -0.17646 | **-0.21767** | | **-0.26236** | **-0.04936** |
| C2WI9 | **-13.692** | -0.42993 | -0.72294 | | -0.45814 | -0.10869 |
| C2WI10 | **22.285** | -0.40929 | -1.61994 | | 0.029479 | 0.362594 |
| C2WI11 | **-4.893** | 0.685311 | -0.1255 | | 0.608359 | 1.573073 |
| C2WI12 | **-9.893** | 0.761902 | **0.486868** | | **0.443505** | **1.355333** |
| C2WI13 | **-18.261** | 0.903146 | 0.383667 | | 0.561589 | 1.76418 |
| C2WI14 | **-11.906** | 0.123052 | -0.09047 | | 0.018601 | 0.44103 |
| C2WI15 | **-13.423** | 0.317669 | -0.28261 | | 0.244055 | 0.991565 |
| C2WI16 | **20.678** | 0.591228 | **0.285019** | | **1.108938** | **0.379727** |
| C2WI17 | **-0.94** | -0.57794 | -1.77692 | | -0.66021 | 0.703311 |

*Bold indicates statistically significant misfit
