# Supplementary material for: Using Rasch analysis to assess the latent construct of the Capacity to Work Index in a Swedish working population sample
Source: Eur J Public Health. 2025 Jan 17;35(3):528–33. doi: 10.1093/eurpub/ckaf001 (PMC12187450; doi:10.1093/eurpub/ckaf001)
Supplement: ckaf001_Supplementary_Data [file ckaf001_supplementary_data.zip › ckaf001_Supplementary_Data/ejph-2024-06-om-0393-File008.docx]

Supplementary file 3.

Table 1. Fit Residuals, location, and threshold values from the C2WI7 analysis containing 7 items, subsample 1. n=1000.

|  |  |  | **Thresholds** | | |
| --- | --- | --- | --- | --- | --- |
| **Item** | **Fit Residuals*** | **Location** | **1*** | **2*** | **3*** |
| C2WI3 | 1.289 | -0.21018 | -1.23314 | -0.12222 | 0.724823 |
| C2WI8 | **-5.554** | -0.21635 | **-0.22123** | **-0.34572** | **-0.08209** |
| C2WI9 | **-3.442** | -0.3978 | -0.64959 | -0.40785 | -0.13597 |
| C2WI10 | **3.848** | -0.41942 | -1.59922 | 0.041567 | 0.29938 |
| C2WI11 | -1.417 | 0.584155 | -0.25096 | 0.718754 | 1.284675 |
| C2WI14 | **-2.625** | 0.11203 | -0.13597 | 0.072665 | 0.399396 |
| C2WI16 | **2.549** | 0.547569 | **0.337531** | **0.835932** | **0.469244** |

*Bold indicates statistically significant misfit

Table 2. Fit Residuals, location, and threshold values from the C2WI7 analysis containing 7 items, subsample 2. n=1000.

|  |  |  | **Thresholds** | | | |
| --- | --- | --- | --- | --- | --- | --- |
| **Item** | **Fit Residuals*** | | **Location** | **1*** | **2*** | **3*** |
| C2WI3 | 0.139 | | -0.21311 | -1.40276 | -0.11169 | 0.875137 |
| C2WI8 | **-3.658** | | -0.10908 | -0.21175 | -0.15792 | 0.042435 |
| C2WI9 | **-4.531** | | -0.42525 | -0.75628 | -0.43846 | -0.08101 |
| C2WI10 | **6.094** | | -0.48183 | -1.73409 | -0.06497 | 0.35358 |
| C2WI11 | -0.339 | | 0.556558 | -0.23223 | 0.528293 | 1.373611 |
| C2WI14 | **-2.869** | | 0.069064 | -0.09756 | -0.07479 | 0.379534 |
| C2WI16 | **1.601** | | 0.603638 | **0.387214** | **1.074234** | **0.349466** |

*Bold indicates statistically significant misfit

Table 3. Fit Residuals, location, and threshold values from the C2WI7 analysis containing 7 items, subsample 3. n=800.

|  |  |  | **Thresholds** | | | |
| --- | --- | --- | --- | --- | --- | --- |
| **Item** | **Fit Residuals*** | | **Location** | **1*** | **2*** | **3*** |
| C2WI3 | 0.88 | | -0.14321 | -1.152 | -0.15598 | 0.87834 |
| C2WI8 | **-4.605** | | -0.18926 | -0.25444 | -0.17071 | -0.14264 |
| C2WI9 | **-3.011** | | -0.50642 | -0.86816 | -0.32695 | -0.32416 |
| C2WI10 | 3.714 | | -0.41141 | -1.60379 | -0.09905 | 0.468612 |
| C2WI11 | -0.305 | | 0.576928 | -0.14528 | 0.609752 | 1.266307 |
| C2WI14 | **-2.804** | | 0.086903 | -0.19023 | 0.035492 | 0.415449 |
| C2WI16 | **2.966** | | 0.586476 | **0.293425** | **1.007841** | **0.458162** |

*Bold indicates statistically significant misfit

Table 4. Fit Residuals, location, and threshold values from the C2WI7 analysis containing 7 items, subsample 4. n=800.

|  |  |  | **Thresholds** | | | |
| --- | --- | --- | --- | --- | --- | --- |
| **Item** | **Fit Residuals*** | | **Location** | **1*** | **2*** | **3*** |
| C2WI3 | 0.753 | | -0.1861 | -1.22766 | -0.14915 | 0.818512 |
| C2WI8 | **-3.366** | | -0.20815 | **-0.02116** | **-0.3071** | **-0.29618** |
| C2WI9 | **-4.682** | | -0.43307 | -0.75314 | -0.62658 | 0.080505 |
| C2WI10 | **4.444** | | -0.43939 | -1.67926 | 0.058967 | 0.302129 |
| C2WI11 | -0.418 | | 0.585423 | -0.05989 | 0.737273 | 1.078888 |
| C2WI14 | -2.233 | | 0.078396 | **0.004672** | **-0.00703** | **0.237545** |
| C2WI16 | **1.011** | | 0.602882 | **0.356633** | **0.98435** | **0.467663** |

*Bold indicates statistically significant misfit

Table 5. Fit Residuals, location, and threshold values from the C2WI7 analysis containing 7 items, subsample 5. n=500.

|  |  |  | **Thresholds** | | | |
| --- | --- | --- | --- | --- | --- | --- |
| **Item** | **Fit Residuals*** | | **Location** | **1*** | **2*** | **3*** |
| C2WI3 | 0.134 | | -0.15941 | -1.1341 | -0.08812 | 0.743985 |
| C2WI8 | **-2.072** | | -0.20682 | -0.32907 | -0.22417 | -0.06721 |
| C2WI9 | **-3.165** | | -0.43732 | -0.89862 | -0.37906 | -0.03426 |
| C2WI10 | 3.425 | | -0.55333 | **-1.77887** | **0.133099** | **-0.01423** |
| C2WI11 | -0.086 | | 0.876919 | -0.15915 | 0.74833 | 2.041577 |
| C2WI14 | -1.393 | | 0.057415 | -0.21021 | 0.190549 | 0.191902 |
| C2WI16 | **2.237** | | 0.422547 | **0.304504** | **1.011881** | **-0.04875** |

*Bold indicates statistically significant misfit

Table 6. Fit Residuals, location, and threshold values from the C2WI7 analysis containing 7 items, subsample 6. Total sample N=8201.

|  |  |  | **Thresholds** | | |
| --- | --- | --- | --- | --- | --- |
| **Item** | **Fit Residuals*** | **Location** | **1*** | **2*** | **3*** |
| C2WI3 | **2.562** | -0.21394 | -1.16869 | -0.23924 | 0.766121 |
| C2WI8 | **-12.985** | -0.19115 | -0.2294 | -0.26302 | -0.08103 |
| C2WI9 | **-12.443** | -0.45591 | -0.78858 | -0.46808 | -0.11108 |
| C2WI10 | **13.273** | -0.43641 | -1.68807 | 0.025783 | 0.353063 |
| C2WI11 | **-2.141** | 0.639732 | -0.13365 | 0.59002 | 1.462829 |
| C2WI14 | **-8.415** | 0.103501 | -0.09308 | 0.007296 | 0.396284 |
| C2WI16 | **7.361** | 0.554171 | 0.278483 | 1.065643 | 0.318386 |

*Bold indicates statistically significant misfit
