# Supplementary material for: Using Rasch analysis to assess the latent construct of the Capacity to Work Index in a Swedish working population sample
Source: Eur J Public Health. 2025 Jan 17;35(3):528–33. doi: 10.1093/eurpub/ckaf001 (PMC12187450; doi:10.1093/eurpub/ckaf001)
Supplement: ckaf001_Supplementary_Data [file ckaf001_supplementary_data.zip › ckaf001_Supplementary_Data/ejph-2024-06-om-0393-File011.docx]

Supplementary file 6.

Table 1. Residual correlation matrix, analysis with 17 items, random sample 1, n=1000, bold indicate values above the critical value for local independence (in this analysis values >0.15).

| **Item** | **C2WI1** | **C2WI2** | **C2WI3** | **C2WI4** | **C2WI5** | **C2WI6** | **C2WI7** | **C2WI8** | **C2WI9** | **C2WI10** | **C2WI11** | **C2WI12** | **C2WI13** | **C2WI14** | **C2WI15** | **C2WI16** | **C2WI17** |
| --- | --- | --- | --- | --- | --- | --- | --- | --- | --- | --- | --- | --- | --- | --- | --- | --- | --- |
| **C2WI1** |  |  |  |  |  |  |  |  |  |  |  |  |  |  |  |  |  |
| **C2WI2** | 0,06 |  |  |  |  |  |  |  |  |  |  |  |  |  |  |  |  |
| **C2WI3** | -0,02 | **0,18** |  |  |  |  |  |  |  |  |  |  |  |  |  |  |  |
| **C2WI4** | 0,06 | -0,28 | -0,12 |  |  |  |  |  |  |  |  |  |  |  |  |  |  |
| **C2WI5** | -0,11 | 0,01 | 0,04 | -0,16 |  |  |  |  |  |  |  |  |  |  |  |  |  |
| **C2WI6** | -0,07 | -0,08 | -0,02 | -0,09 | **0,26** |  |  |  |  |  |  |  |  |  |  |  |  |
| **C2WI7** | -0,15 | 0,00 | -0,13 | -0,22 | 0,01 | -0,01 |  |  |  |  |  |  |  |  |  |  |  |
| **C2WI8** | -0,15 | -0,05 | -0,19 | -0,23 | -0,06 | -0,13 | **0,17** |  |  |  |  |  |  |  |  |  |  |
| **C2WI9** | -0,11 | -0,07 | -0,13 | -0,17 | -0,17 | -0,12 | 0,13 | **0,23** |  |  |  |  |  |  |  |  |  |
| **C2WI10** | -0,07 | -0,10 | -0,12 | -0,07 | -0,13 | -0,15 | -0,06 | -0,08 | -0,16 |  |  |  |  |  |  |  |  |
| **C2WI11** | -0,10 | 0,07 | 0,12 | -0,15 | -0,02 | 0,08 | -0,09 | -0,15 | -0,10 | -0,10 |  |  |  |  |  |  |  |
| **C2WI12** | -0,09 | -0,04 | -0,05 | -0,11 | -0,01 | 0,02 | 0,03 | -0,07 | -0,12 | 0,01 | 0,08 |  |  |  |  |  |  |
| **C2WI13** | -0,10 | 0,11 | 0,05 | -0,25 | -0,05 | -0,03 | 0,11 | -0,04 | -0,08 | -0,05 | 0,06 | **0,22** |  |  |  |  |  |
| **C2WI14** | -0,09 | -0,05 | -0,15 | -0,12 | -0,14 | -0,06 | 0,04 | 0,02 | 0,07 | -0,10 | -0,06 | 0,02 | 0,13 |  |  |  |  |
| **C2WI15** | -0,10 | -0,01 | -0,15 | -0,19 | -0,13 | -0,08 | -0,03 | **0,18** | 0,15 | -0,08 | -0,07 | -0,06 | -0,02 | 0,07 |  |  |  |
| **C2WI16** | -0,06 | -0,10 | -0,11 | -0,06 | 0,06 | -0,04 | -0,11 | -0,04 | -0,09 | 0,13 | -0,10 | -0,08 | -0,14 | -0,10 | -0,06 |  |  |
| **C2WI17** | -0,08 | -0,09 | 0,01 | -0,10 | 0,06 | -0,03 | -0,10 | -0,04 | -0,06 | -0,13 | -0,06 | -0,21 | -0,11 | -0,08 | -0,03 | -0,04 |  |

Table 2. Residual correlation matrix, analysis with 17 items, random sample 2, n=1000, bold indicate values above the critical value for local independence (in this analysis values >0.15).

| **Item** | **C2WI1** | **C2WI2** | **C2WI3** | **C2WI4** | **C2WI5** | **C2WI6** | **C2WI7** | **C2WI8** | **C2WI9** | **C2WI10** | **C2WI11** | **C2WI12** | **C2WI13** | **C2WI14** | **C2WI15** | **C2WI16** | **C2WI17** |
| --- | --- | --- | --- | --- | --- | --- | --- | --- | --- | --- | --- | --- | --- | --- | --- | --- | --- |
| **C2WI1** |  |  |  |  |  |  |  |  |  |  |  |  |  |  |  |  |  |
| **C2WI2** | 0,018 |  |  |  |  |  |  |  |  |  |  |  |  |  |  |  |  |
| **C2WI3** | -0,001 | **0,181** |  |  |  |  |  |  |  |  |  |  |  |  |  |  |  |
| **C2WI4** | 0,011 | -0,294 | -0,183 |  |  |  |  |  |  |  |  |  |  |  |  |  |  |
| **C2WI5** | -0,126 | 0,051 | 0,043 | -0,192 |  |  |  |  |  |  |  |  |  |  |  |  |  |
| **C2WI6** | -0,11 | -0,065 | -0,068 | -0,054 | **0,231** |  |  |  |  |  |  |  |  |  |  |  |  |
| **C2WI7** | -0,149 | 0,028 | -0,144 | -0,264 | 0,058 | 0,033 |  |  |  |  |  |  |  |  |  |  |  |
| **C2WI8** | -0,105 | -0,086 | -0,122 | -0,174 | -0,057 | -0,104 | **0,169** |  |  |  |  |  |  |  |  |  |  |
| **C2WI9** | -0,101 | -0,065 | -0,072 | -0,191 | -0,084 | -0,065 | 0,08 | **0,245** |  |  |  |  |  |  |  |  |  |
| **C2WI10** | -0,027 | -0,132 | -0,154 | -0,002 | -0,175 | -0,229 | -0,105 | -0,103 | -0,125 |  |  |  |  |  |  |  |  |
| **C2WI11** | -0,058 | 0,083 | 0,118 | -0,167 | -0,046 | -0,013 | -0,081 | -0,13 | -0,102 | -0,144 |  |  |  |  |  |  |  |
| **C2WI12** | -0,089 | -0,006 | -0,083 | -0,149 | -0,044 | -0,025 | 0,09 | -0,098 | -0,116 | -0,018 | 0,052 |  |  |  |  |  |  |
| **C2WI13** | -0,145 | 0,101 | 0,005 | -0,31 | 0,032 | -0,054 | 0,14 | -0,041 | -0,119 | -0,105 | 0,087 | **0,295** |  |  |  |  |  |
| **C2WI14** | -0,079 | 0,055 | -0,097 | -0,174 | -0,09 | -0,076 | 0,074 | -0,029 | -0,022 | -0,096 | -0,047 | 0,064 | 0,131 |  |  |  |  |
| **C2WI15** | -0,085 | -0,013 | -0,095 | -0,202 | -0,059 | -0,12 | -0,025 | 0,149 | 0,117 | -0,134 | 0,013 | -0,062 | 0,064 | 0,079 |  |  |  |
| **C2WI16** | -0,094 | -0,144 | -0,024 | -0,014 | 0,011 | -0,086 | -0,105 | -0,08 | -0,04 | **0,191** | -0,081 | -0,123 | -0,131 | -0,121 | -0,111 |  |  |
| **C2WI17** | -0,12 | -0,047 | 0,013 | -0,068 | 0,08 | 0,069 | -0,065 | -0,06 | -0,002 | -0,191 | -0,061 | -0,096 | -0,053 | -0,072 | 0,006 | -0,117 |  |

Table 3. Residual correlation matrix, analysis with 17 items, random sample 3, n=800, bold indicate values above the critical value for local independence (in this analysis values >0.15).

| **Item** | **C2WI1** | **C2WI2** | **C2WI3** | **C2WI4** | **C2WI5** | **C2WI6** | **C2WI7** | **C2WI8** | **C2WI9** | **C2WI10** | **C2WI11** | **C2WI12** | **C2WI13** | **C2WI14** | **C2WI15** | **C2WI16** | **C2WI17** |
| --- | --- | --- | --- | --- | --- | --- | --- | --- | --- | --- | --- | --- | --- | --- | --- | --- | --- |
| **C2WI1** |  |  |  |  |  |  |  |  |  |  |  |  |  |  |  |  |  |
| **C2WI2** | 0,067 |  |  |  |  |  |  |  |  |  |  |  |  |  |  |  |  |
| **C2WI3** | 0,003 | **0,234** |  |  |  |  |  |  |  |  |  |  |  |  |  |  |  |
| **C2WI4** | 0,086 | -0,286 | -0,15 |  |  |  |  |  |  |  |  |  |  |  |  |  |  |
| **C2WI5** | -0,091 | -0,014 | -0,095 | -0,154 |  |  |  |  |  |  |  |  |  |  |  |  |  |
| **C2WI6** | -0,111 | -0,069 | -0,063 | -0,092 | **0,226** |  |  |  |  |  |  |  |  |  |  |  |  |
| **C2WI7** | -0,13 | 0,033 | -0,13 | -0,291 | 0,046 | 0,099 |  |  |  |  |  |  |  |  |  |  |  |
| **C2WI8** | -0,1 | -0,012 | -0,122 | -0,218 | 0,027 | -0,104 | 0,108 |  |  |  |  |  |  |  |  |  |  |
| **C2WI9** | -0,087 | -0,051 | -0,065 | -0,207 | -0,156 | -0,118 | 0,05 | **0,181** |  |  |  |  |  |  |  |  |  |
| **C2WI10** | -0,134 | -0,172 | -0,151 | -0,02 | -0,133 | -0,128 | -0,058 | -0,058 | -0,148 |  |  |  |  |  |  |  |  |
| **C2WI11** | -0,03 | 0,076 | 0,06 | -0,168 | -0,079 | -0,008 | -0,082 | -0,137 | -0,068 | -0,081 |  |  |  |  |  |  |  |
| **C2WI12** | -0,124 | 0,003 | -0,069 | -0,159 | -0,082 | -0,03 | 0,061 | -0,159 | -0,108 | 0,068 | 0,09 |  |  |  |  |  |  |
| **C2WI13** | -0,108 | 0,128 | 0,046 | -0,297 | -0,068 | -0,082 | 0,055 | -0,027 | -0,088 | -0,017 | 0,097 | **0,26** |  |  |  |  |  |
| **C2WI14** | -0,117 | -0,142 | -0,136 | -0,187 | -0,031 | -0,033 | 0,082 | 0,013 | 0,057 | -0,008 | -0,067 | 0,096 | 0,102 |  |  |  |  |
| **C2WI15** | -0,133 | 0,02 | -0,068 | -0,313 | 0,024 | -0,12 | 0,048 | 0,142 | 0,107 | -0,124 | 0 | -0,018 | 0,078 | 0,082 |  |  |  |
| **C2WI16** | -0,056 | -0,076 | -0,127 | 0,038 | 0,013 | -0,071 | -0,095 | -0,066 | -0,101 | 0,089 | -0,055 | -0,096 | -0,154 | -0,147 | -0,048 |  |  |
| **C2WI17** | -0,123 | -0,058 | 0,09 | -0,074 | 0,096 | 0,023 | -0,075 | -0,017 | 0,026 | -0,198 | -0,136 | -0,177 | -0,101 | -0,16 | 0,009 | -0,027 |  |

Table 4. Residual correlation matrix, analysis with 17 items, random sample 4, n=800, bold indicate values above the critical value for local independence (in this analysis values >0.15).

| **Item** | **C2WI1** | **C2WI2** | **C2WI3** | **C2WI4** | **C2WI5** | **C2WI6** | **C2WI7** | **C2WI8** | **C2WI9** | **C2WI10** | **C2WI11** | **C2WI12** | **C2WI13** | **C2WI14** | **C2WI15** | **C2WI16** | **C2WI17** |
| --- | --- | --- | --- | --- | --- | --- | --- | --- | --- | --- | --- | --- | --- | --- | --- | --- | --- |
| **C2WI1** |  |  |  |  |  |  |  |  |  |  |  |  |  |  |  |  |  |
| **C2WI2** | 0,067 |  |  |  |  |  |  |  |  |  |  |  |  |  |  |  |  |
| **C2WI3** | 0,003 | **0,234** |  |  |  |  |  |  |  |  |  |  |  |  |  |  |  |
| **C2WI4** | 0,086 | -0,286 | -0,15 |  |  |  |  |  |  |  |  |  |  |  |  |  |  |
| **C2WI5** | -0,091 | -0,014 | -0,095 | -0,154 |  |  |  |  |  |  |  |  |  |  |  |  |  |
| **C2WI6** | -0,111 | -0,069 | -0,063 | -0,092 | **0,226** |  |  |  |  |  |  |  |  |  |  |  |  |
| **C2WI7** | -0,13 | 0,033 | -0,13 | -0,291 | 0,046 | 0,099 |  |  |  |  |  |  |  |  |  |  |  |
| **C2WI8** | -0,1 | -0,012 | -0,122 | -0,218 | 0,027 | -0,104 | 0,108 |  |  |  |  |  |  |  |  |  |  |
| **C2WI9** | -0,087 | -0,051 | -0,065 | -0,207 | -0,156 | -0,118 | 0,05 | **0,181** |  |  |  |  |  |  |  |  |  |
| **C2WI10** | -0,134 | -0,172 | -0,151 | -0,02 | -0,133 | -0,128 | -0,058 | -0,058 | -0,148 |  |  |  |  |  |  |  |  |
| **C2WI11** | -0,03 | 0,076 | 0,06 | -0,168 | -0,079 | -0,008 | -0,082 | -0,137 | -0,068 | -0,081 |  |  |  |  |  |  |  |
| **C2WI12** | -0,124 | 0,003 | -0,069 | -0,159 | -0,082 | -0,03 | 0,061 | -0,159 | -0,108 | 0,068 | 0,09 |  |  |  |  |  |  |
| **C2WI13** | -0,108 | 0,128 | 0,046 | -0,297 | -0,068 | -0,082 | 0,055 | -0,027 | -0,088 | -0,017 | 0,097 | **0,26** |  |  |  |  |  |
| **C2WI14** | -0,117 | -0,142 | -0,136 | -0,187 | -0,031 | -0,033 | 0,082 | 0,013 | 0,057 | -0,008 | -0,067 | 0,096 | 0,102 |  |  |  |  |
| **C2WI15** | -0,133 | 0,02 | -0,068 | -0,313 | 0,024 | -0,12 | 0,048 | 0,142 | 0,107 | -0,124 | 0 | -0,018 | 0,078 | 0,082 |  |  |  |
| **C2WI16** | -0,056 | -0,076 | -0,127 | 0,038 | 0,013 | -0,071 | -0,095 | -0,066 | -0,101 | 0,089 | -0,055 | -0,096 | -0,154 | -0,147 | -0,048 |  |  |
| **C2WI17** | -0,123 | -0,058 | 0,09 | -0,074 | 0,096 | 0,023 | -0,075 | -0,017 | 0,026 | -0,198 | -0,136 | -0,177 | -0,101 | -0,16 | 0,009 | -0,027 |  |

Table 5. Residual correlation matrix, analysis with 17 items, random sample 5, n=500, bold indicate values above the critical value for local independence (in this analysis values >0.15).

| **Item** | **C2WI1** | **C2WI2** | **C2WI3** | **C2WI4** | **C2WI5** | **C2WI6** | **C2WI7** | **C2WI8** | **C2WI9** | **C2WI10** | **C2WI11** | **C2WI12** | **C2WI13** | **C2WI14** | **C2WI15** | **C2WI16** | **C2WI17** |
| --- | --- | --- | --- | --- | --- | --- | --- | --- | --- | --- | --- | --- | --- | --- | --- | --- | --- |
| **C2WI1** |  |  |  |  |  |  |  |  |  |  |  |  |  |  |  |  |  |
| **C2WI2** | 0,098 |  |  |  |  |  |  |  |  |  |  |  |  |  |  |  |  |
| **C2WI3** | -0,024 | **0,182** |  |  |  |  |  |  |  |  |  |  |  |  |  |  |  |
| **C2WI4** | -0,061 | -0,276 | -0,164 |  |  |  |  |  |  |  |  |  |  |  |  |  |  |
| **C2WI5** | -0,137 | -0,034 | -0,016 | -0,171 |  |  |  |  |  |  |  |  |  |  |  |  |  |
| **C2WI6** | -0,079 | 0,028 | 0,021 | -0,176 | **0,244** |  |  |  |  |  |  |  |  |  |  |  |  |
| **C2WI7** | -0,16 | -0,022 | -0,032 | -0,253 | 0,062 | 0,14 |  |  |  |  |  |  |  |  |  |  |  |
| **C2WI8** | -0,123 | -0,101 | -0,113 | -0,135 | 0,003 | -0,067 | 0,149 |  |  |  |  |  |  |  |  |  |  |
| **C2WI9** | -0,019 | 0,002 | -0,096 | -0,201 | -0,067 | -0,118 | 0,018 | **0,213** |  |  |  |  |  |  |  |  |  |
| **C2WI10** | -0,139 | -0,224 | -0,16 | 0,014 | -0,123 | -0,168 | -0,063 | -0,127 | -0,144 |  |  |  |  |  |  |  |  |
| **C2WI11** | -0,05 | **0,162** | 0,132 | -0,175 | 0,002 | 0,061 | -0,103 | -0,152 | -0,102 | -0,096 |  |  |  |  |  |  |  |
| **C2WI12** | -0,052 | -0,086 | -0,128 | -0,089 | -0,012 | 0,076 | 0,009 | -0,154 | -0,125 | 0,043 | 0,052 |  |  |  |  |  |  |
| **C2WI13** | -0,025 | 0,15 | 0,032 | -0,284 | 0,064 | -0,082 | 0,046 | -0,036 | -0,063 | -0,109 | 0,072 | **0,193** |  |  |  |  |  |
| **C2WI14** | 0,036 | -0,016 | -0,158 | -0,132 | -0,159 | -0,087 | 0,08 | -0,038 | 0,045 | -0,097 | -0,105 | -0,004 | -0,036 |  |  |  |  |
| **C2WI15** | -0,019 | 0,019 | -0,082 | -0,181 | -0,06 | -0,151 | -0,11 | 0,096 | 0,109 | -0,089 | -0,034 | -0,095 | 0,073 | 0,112 |  |  |  |
| **C2WI16** | -0,041 | -0,186 | -0,088 | 0,015 | -0,097 | -0,11 | -0,097 | -0,075 | -0,076 | **0,237** | -0,107 | -0,083 | -0,108 | -0,127 | -0,097 |  |  |
| **C2WI17** | -0,136 | 0,01 | 0,074 | -0,106 | 0,061 | -0,058 | -0,011 | -0,079 | -0,008 | -0,221 | -0,051 | -0,142 | -0,041 | -0,029 | 0,011 | -0,086 |  |

Table 6. Residual correlation matrix, analysis with 17 items, total sample 6, N=8201, bold indicate values above the critical value for local independence (in this analysis values >0.15).

| **Item** | **C2WI1** | **C2WI2** | **C2WI3** | **C2WI4** | **C2WI5** | **C2WI6** | **C2WI7** | **C2WI8** | **C2WI9** | **C2WI10** | **C2WI11** | **C2WI12** | **C2WI13** | **C2WI14** | **C2WI15** | **C2WI16** | **C2WI17** |
| --- | --- | --- | --- | --- | --- | --- | --- | --- | --- | --- | --- | --- | --- | --- | --- | --- | --- |
| **C2WI1** |  |  |  |  |  |  |  |  |  |  |  |  |  |  |  |  |  |
| **C2WI2** | 0,069 |  |  |  |  |  |  |  |  |  |  |  |  |  |  |  |  |
| **C2WI3** | -0,005 | **0,18** |  |  |  |  |  |  |  |  |  |  |  |  |  |  |  |
| **C2WI4** | 0,036 | -0,275 | -0,146 |  |  |  |  |  |  |  |  |  |  |  |  |  |  |
| **C2WI5** | -0,106 | -0,005 | -0,024 | -0,166 |  |  |  |  |  |  |  |  |  |  |  |  |  |
| **C2WI6** | -0,113 | -0,066 | -0,049 | -0,112 | **0,196** |  |  |  |  |  |  |  |  |  |  |  |  |
| **C2WI7** | -0,143 | 0,014 | -0,119 | -0,252 | 0,034 | 0,064 |  |  |  |  |  |  |  |  |  |  |  |
| **C2WI8** | -0,123 | -0,047 | -0,149 | -0,207 | -0,035 | -0,066 | 0,149 |  |  |  |  |  |  |  |  |  |  |
| **C2WI9** | -0,089 | -0,043 | -0,079 | -0,201 | -0,12 | -0,1 | 0,034 | **0,203** |  |  |  |  |  |  |  |  |  |
| **C2WI10** | -0,088 | -0,141 | -0,146 | 0,008 | -0,126 | -0,172 | -0,078 | -0,087 | -0,134 |  |  |  |  |  |  |  |  |
| **C2WI11** | -0,08 | 0,032 | 0,075 | -0,125 | -0,055 | 0,018 | -0,087 | -0,132 | -0,071 | -0,116 |  |  |  |  |  |  |  |
| **C2WI12** | -0,095 | -0,037 | -0,08 | -0,139 | -0,037 | 0,01 | 0,076 | -0,085 | -0,111 | -0,01 | 0,066 |  |  |  |  |  |  |
| **C2WI13** | -0,113 | 0,093 | 0,017 | -0,284 | 0,004 | -0,031 | 0,077 | -0,033 | -0,06 | -0,082 | 0,063 | **0,245** |  |  |  |  |  |
| **C2WI14** | -0,082 | -0,03 | -0,139 | -0,149 | -0,092 | -0,059 | 0,055 | -0,009 | 0,061 | -0,068 | -0,036 | 0,036 | 0,069 |  |  |  |  |
| **C2WI15** | -0,106 | 0,003 | -0,12 | -0,209 | -0,082 | -0,102 | -0,01 | **0,182** | 0,128 | -0,108 | -0,036 | -0,057 | 0,059 | 0,078 |  |  |  |
| **C2WI16** | -0,056 | -0,117 | -0,071 | -0,024 | 0,007 | -0,071 | -0,107 | -0,086 | -0,088 | 0,133 | -0,06 | -0,087 | -0,119 | -0,108 | -0,083 |  |  |
| **C2WI17** | -0,084 | -0,068 | 0,035 | -0,093 | 0,069 | 0,009 | -0,069 | -0,041 | -0,003 | -0,179 | -0,085 | -0,134 | -0,092 | -0,121 | -0,022 | -0,043 |  |
