# Supplementary material for: Using Rasch analysis to assess the latent construct of the Capacity to Work Index in a Swedish working population sample
Source: Eur J Public Health. 2025 Jan 17;35(3):528–33. doi: 10.1093/eurpub/ckaf001 (PMC12187450; doi:10.1093/eurpub/ckaf001)
Supplement: ckaf001_Supplementary_Data [file ckaf001_supplementary_data.zip › ckaf001_Supplementary_Data/ejph-2024-06-om-0393-File012.docx]

Supplementary file 7.

Table 1. Residual correlation matrix, analysis with 7 items, random sample 1, n=1000, bold indicate values above the critical value for local independence (in this analysis values >0.15).

| \| **Item** \| **C2WI3** \| **C2WI8** \| **C2WI9** \| **C2WI10** \| **C2WI11** \| **C2WI14** \| **C2WI16** \| \| --- \| --- \| --- \| --- \| --- \| --- \| --- \| --- \| \| **C2WI3** \|  \|  \|  \|  \|  \|  \|  \| \| **C2WI8** \| -0,25 \|  \|  \|  \|  \|  \|  \| \| **C2WI9** \| -0,222 \| **0,139** \|  \|  \|  \|  \|  \| \| **C2WI10** \| -0,223 \| -0,228 \| -0,346 \|  \|  \|  \|  \| \| **C2WI11** \| **0,075** \| -0,199 \| -0,178 \| -0,203 \|  \|  \|  \| \| **C2WI14** \| -0,207 \| -0,06 \| -0,026 \| -0,202 \| -0,104 \|  \|  \| \| **C2WI16** \| -0,187 \| -0,165 \| -0,248 \| -0,044 \| -0,163 \| -0,185 \|  \| |
| --- | --- | --- | --- | --- | --- | --- | --- | --- | --- | --- | --- | --- | --- | --- | --- | --- | --- | --- | --- | --- | --- | --- | --- | --- | --- | --- | --- | --- | --- | --- | --- | --- | --- | --- | --- | --- | --- | --- | --- | --- | --- | --- | --- | --- | --- | --- | --- | --- | --- | --- | --- | --- | --- | --- | --- | --- | --- | --- | --- | --- | --- | --- | --- | --- |

Table 2. Residual correlation matrix, analysis with 7 items, random sample 2, n=1000, bold indicate values above the critical value for local independence (in this analysis values >0.15).

| **Item** | **C2WI3** | **C2WI8** | **C2WI9** | **C2WI10** | **C2WI11** | **C2WI14** | **C2WI16** |
| --- | --- | --- | --- | --- | --- | --- | --- |
| **C2WI3** |  |  |  |  |  |  |  |
| **C2WI8** | -0,195 |  |  |  |  |  |  |
| **C2WI9** | -0,159 | **0,143** |  |  |  |  |  |
| **C2WI10** | -0,313 | -0,268 | -0,33 |  |  |  |  |
| **C2WI11** | **0,059** | -0,189 | -0,182 | -0,263 |  |  |  |
| **C2WI14** | -0,152 | -0,075 | -0,089 | -0,205 | -0,076 |  |  |
| **C2WI16** | -0,131 | -0,208 | -0,199 | 0,002 | -0,162 | -0,218 |  |

Table 3. Residual correlation matrix, analysis with 7 items, random sample 3, n=800, bold indicate values above the critical value for local independence (in this analysis values >0.15).

| **Item** | **C2WI3** | **C2WI8** | **C2WI9** | **C2WI10** | **C2WI11** | **C2WI14** | **C2WI16** |
| --- | --- | --- | --- | --- | --- | --- | --- |
| **C2WI3** |  |  |  |  |  |  |  |
| **C2WI8** | -0,182 |  |  |  |  |  |  |
| **C2WI9** | -0,153 | **0,075** |  |  |  |  |  |
| **C2WI10** | -0,283 | -0,215 | -0,368 |  |  |  |  |
| **C2WI11** | 0,013 | -0,213 | -0,158 | -0,203 |  |  |  |
| **C2WI14** | -0,186 | -0,06 | -0,039 | -0,137 | -0,119 |  |  |
| **C2WI16** | -0,189 | -0,172 | -0,233 | -0,045 | -0,123 | -0,23 |  |

Table 4. Residual correlation matrix, analysis with 7 items, random sample 4, n=800, bold indicate values above the critical value for local independence (in this analysis values >0.15).

| **Item** | **C2WI3** | **C2WI8** | **C2WI9** | **C2WI10** | **C2WI11** | **C2WI14** | **C2WI16** |
| --- | --- | --- | --- | --- | --- | --- | --- |
| **C2WI3** |  |  |  |  |  |  |  |
| **C2WI8** | -0,215 |  |  |  |  |  |  |
| **C2WI9** | -0,204 | **0,131** |  |  |  |  |  |
| **C2WI10** | -0,261 | -0,227 | -0,321 |  |  |  |  |
| **C2WI11** | 0,043 | -0,186 | -0,166 | -0,302 |  |  |  |
| **C2WI14** | -0,137 | -0,196 | -0,049 | -0,177 | -0,056 |  |  |
| **C2WI16** | -0,173 | -0,165 | -0,19 | -0,037 | -0,105 | -0,215 |  |

Table 5. Residual correlation matrix, analysis with 7 items, random sample 5, n=500, bold indicate values above the critical value for local independence (in this analysis values >0.15).

| **Item** | **C2WI3** | **C2WI8** | **C2WI9** | **C2WI10** | **C2WI11** | **C2WI14** | **C2WI16** |
| --- | --- | --- | --- | --- | --- | --- | --- |
| **C2WI3** |  |  |  |  |  |  |  |
| **C2WI8** | -0,166 |  |  |  |  |  |  |
| **C2WI9** | -0,158 | **0,121** |  |  |  |  |  |
| **C2WI10** | -0,287 | -0,306 | -0,35 |  |  |  |  |
| **C2WI11** | **0,101** | -0,189 | -0,149 | -0,2 |  |  |  |
| **C2WI14** | -0,199 | -0,098 | -0,007 | -0,195 | -0,118 |  |  |
| **C2WI16** | -0,186 | -0,206 | -0,256 | 0,019 | -0,175 | -0,236 |  |

Table 6. Residual correlation matrix, analysis with 7 items, total sample 6, N=8201, bold indicate values above the critical value for local independence (in this analysis values >0.15).

| **Item** | **C2WI3** | **C2WI8** | **C2WI9** | **C2WI10** | **C2WI11** | **C2WI14** | **C2WI16** |
| --- | --- | --- | --- | --- | --- | --- | --- |
| **C2WI3** |  |  |  |  |  |  |  |
| **C2WI8** | -0,211 |  |  |  |  |  |  |
| **C2WI9** | -0,176 | **0,112** |  |  |  |  |  |
| **C2WI10** | -0,285 | -0,233 | -0,334 |  |  |  |  |
| **C2WI11** | 0,024 | -0,192 | -0,159 | -0,234 |  |  |  |
| **C2WI14** | -0,201 | -0,073 | -0,035 | -0,189 | -0,088 |  |  |
| **C2WI16** | -0,156 | -0,19 | -0,236 | -0,024 | -0,136 | -0,202 |  |
