# Supplementary figures and images for: Using Rasch analysis to assess the latent construct of the Capacity to Work Index in a Swedish working population sample
Source: Eur J Public Health. 2025 Jan 17;35(3):528–33. doi: 10.1093/eurpub/ckaf001 (PMC12187450; doi:10.1093/eurpub/ckaf001)

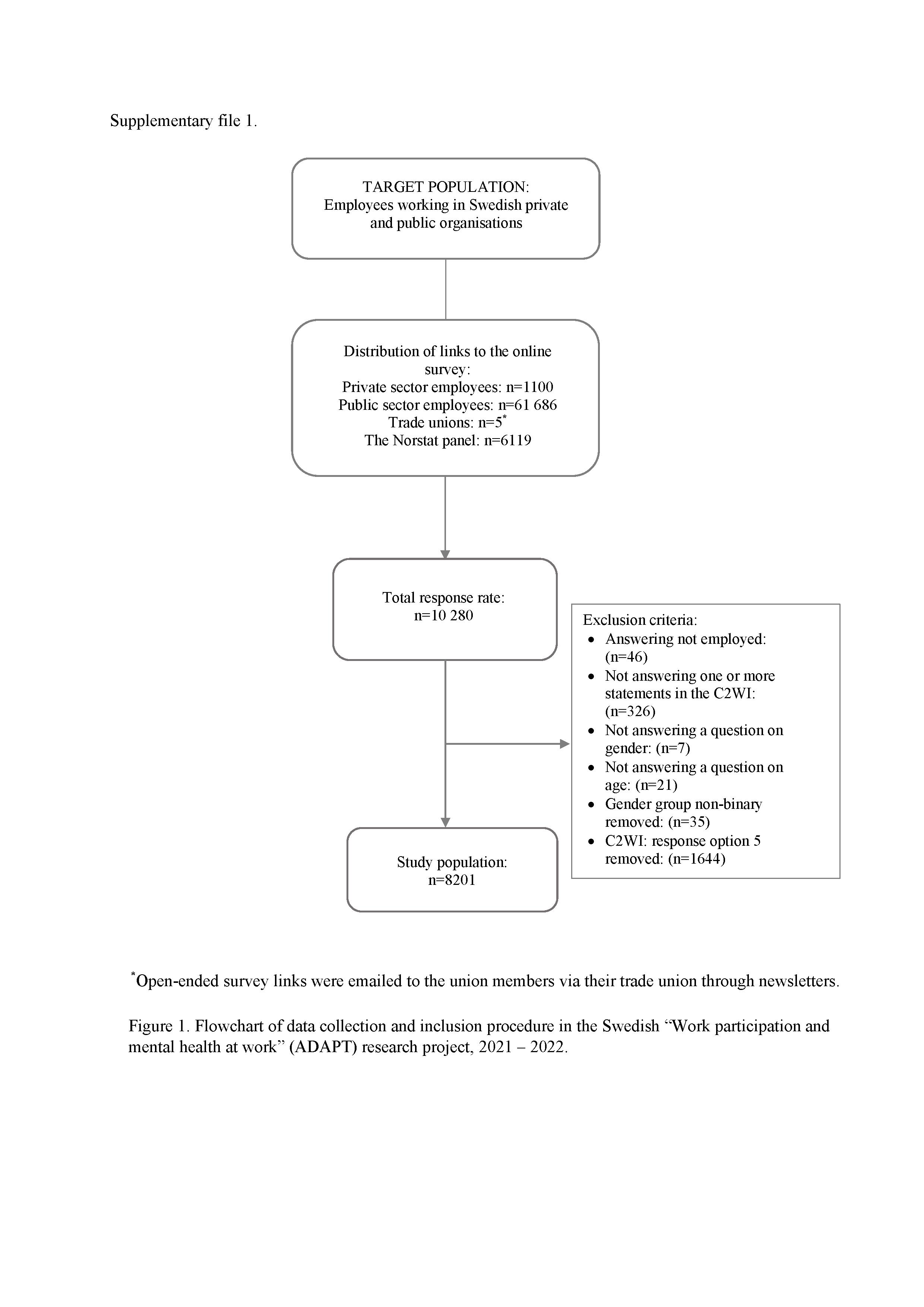

Supplement: ckaf001_Supplementary_Data [file ckaf001_supplementary_data.zip › ckaf001_Supplementary_Data/ejph-2024-06-om-0393-File006.tif]

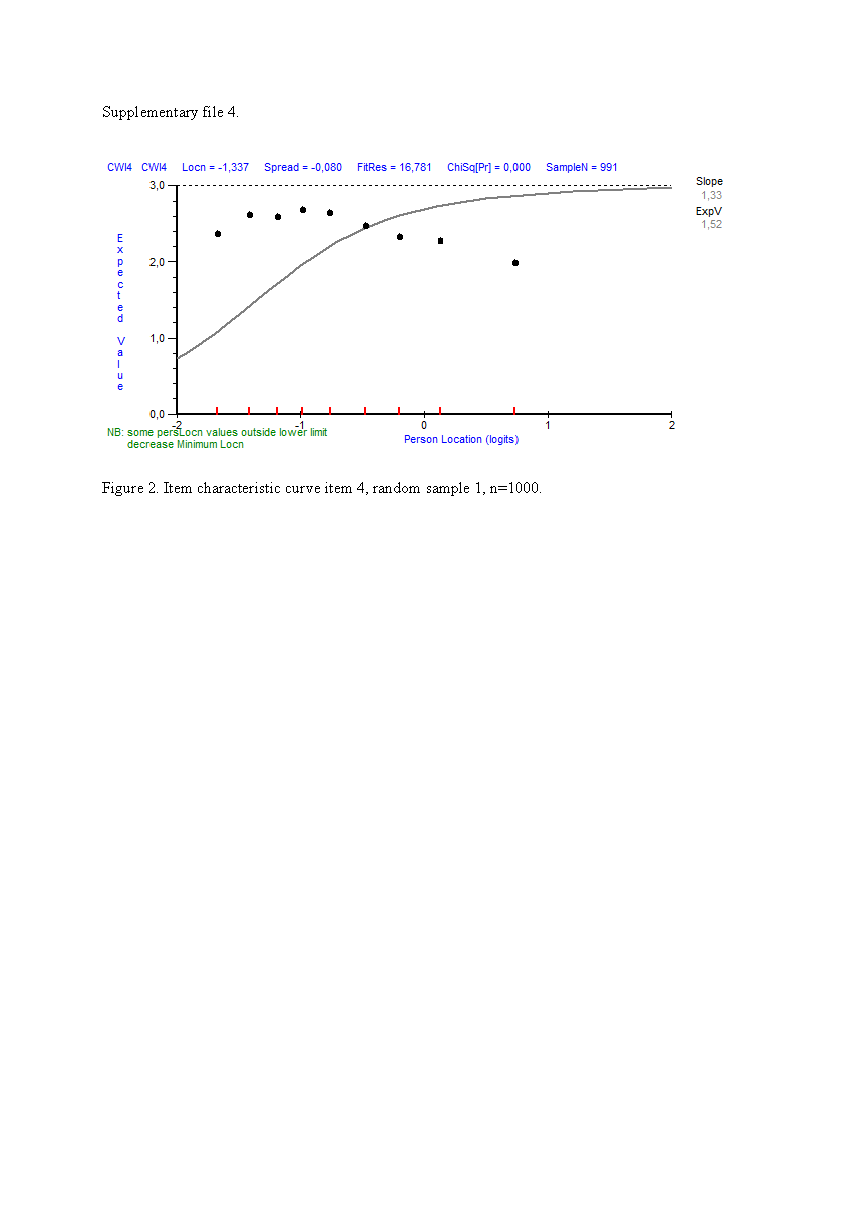

Supplement: ckaf001_Supplementary_Data [file ckaf001_supplementary_data.zip › ckaf001_Supplementary_Data/ejph-2024-06-om-0393-File009.tif]

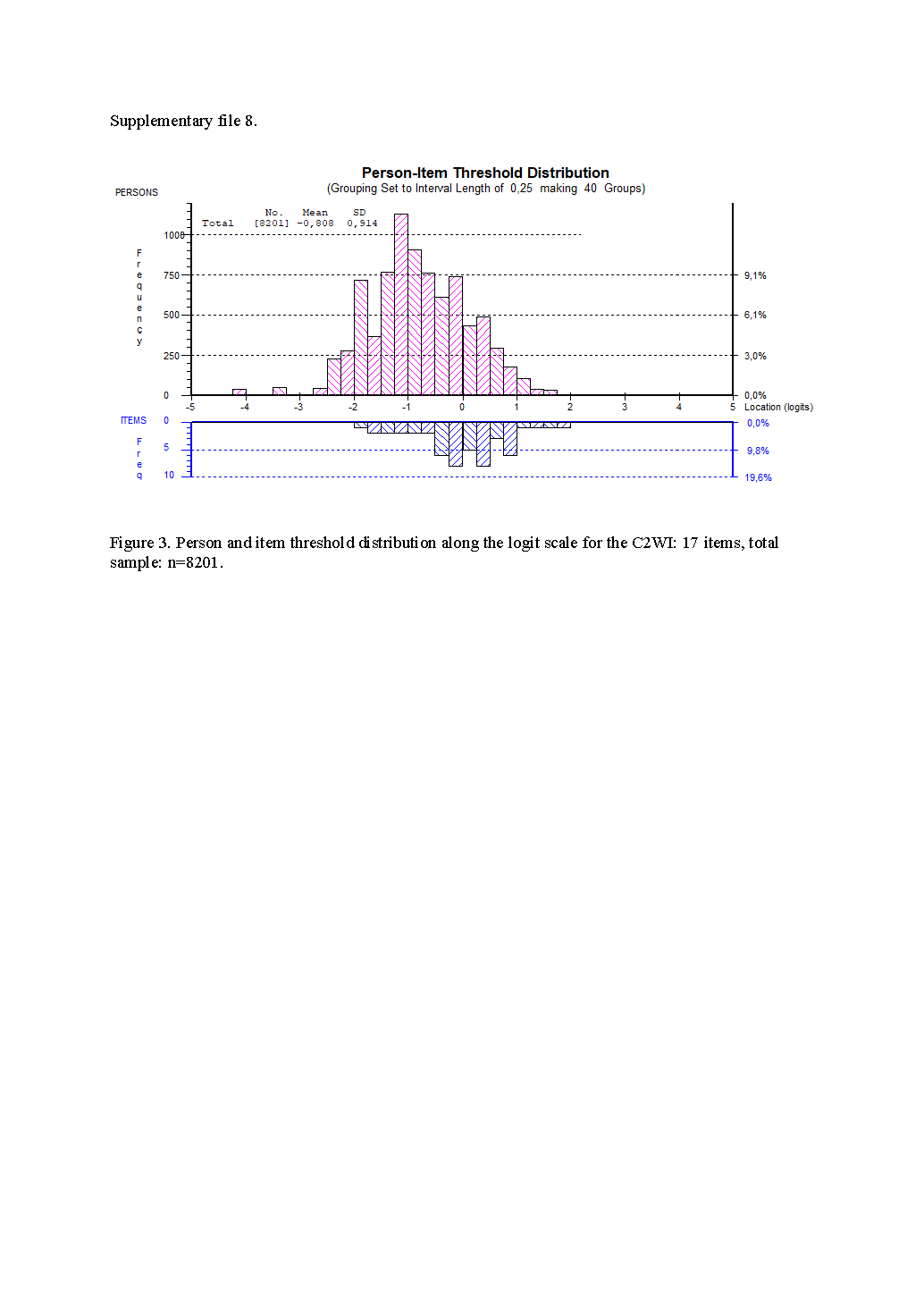

Supplement: ckaf001_Supplementary_Data [file ckaf001_supplementary_data.zip › ckaf001_Supplementary_Data/ejph-2024-06-om-0393-File013.tif]

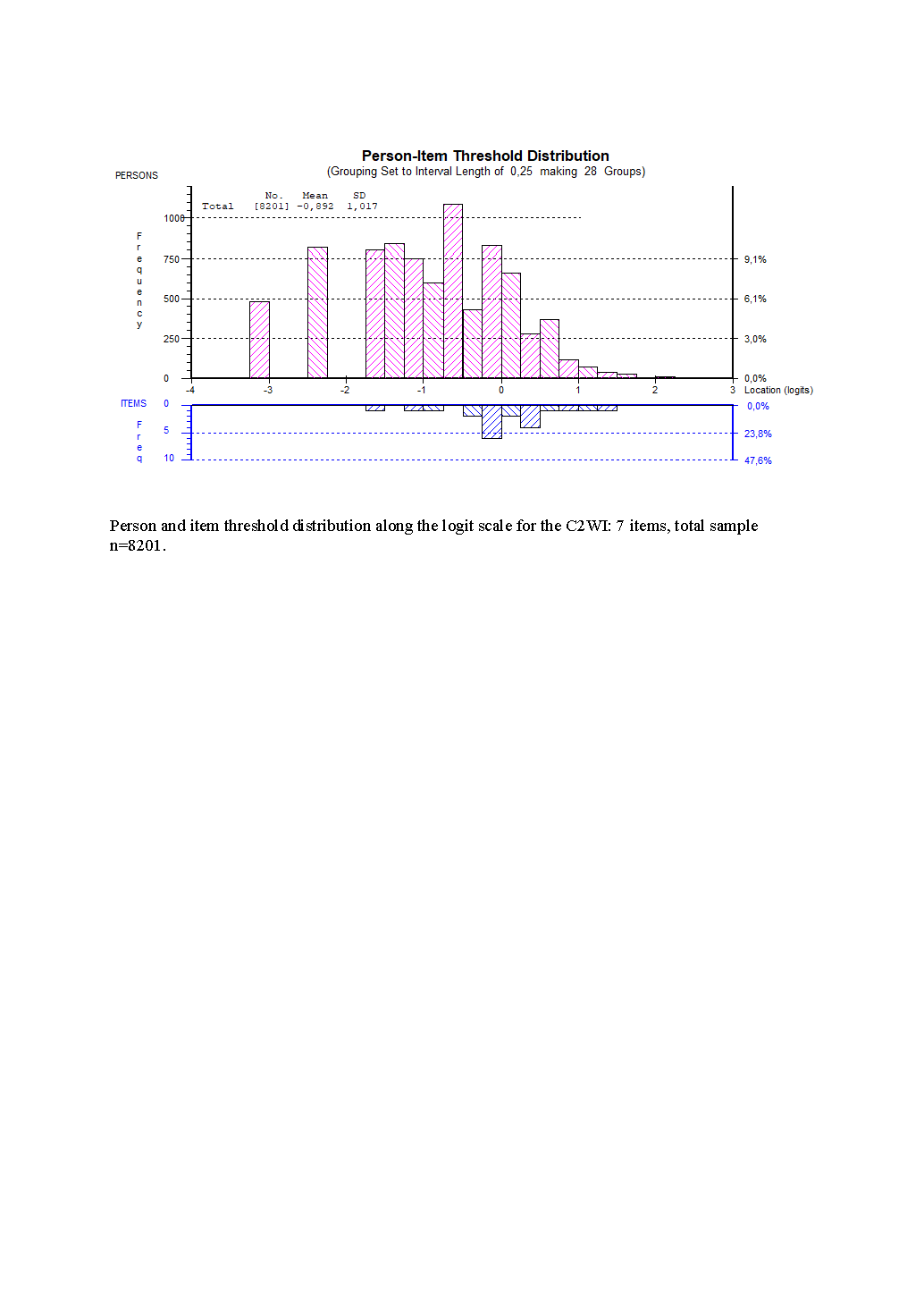

Supplement: ckaf001_Supplementary_Data [file ckaf001_supplementary_data.zip › ckaf001_Supplementary_Data/ejph-2024-06-om-0393-File014.tif]

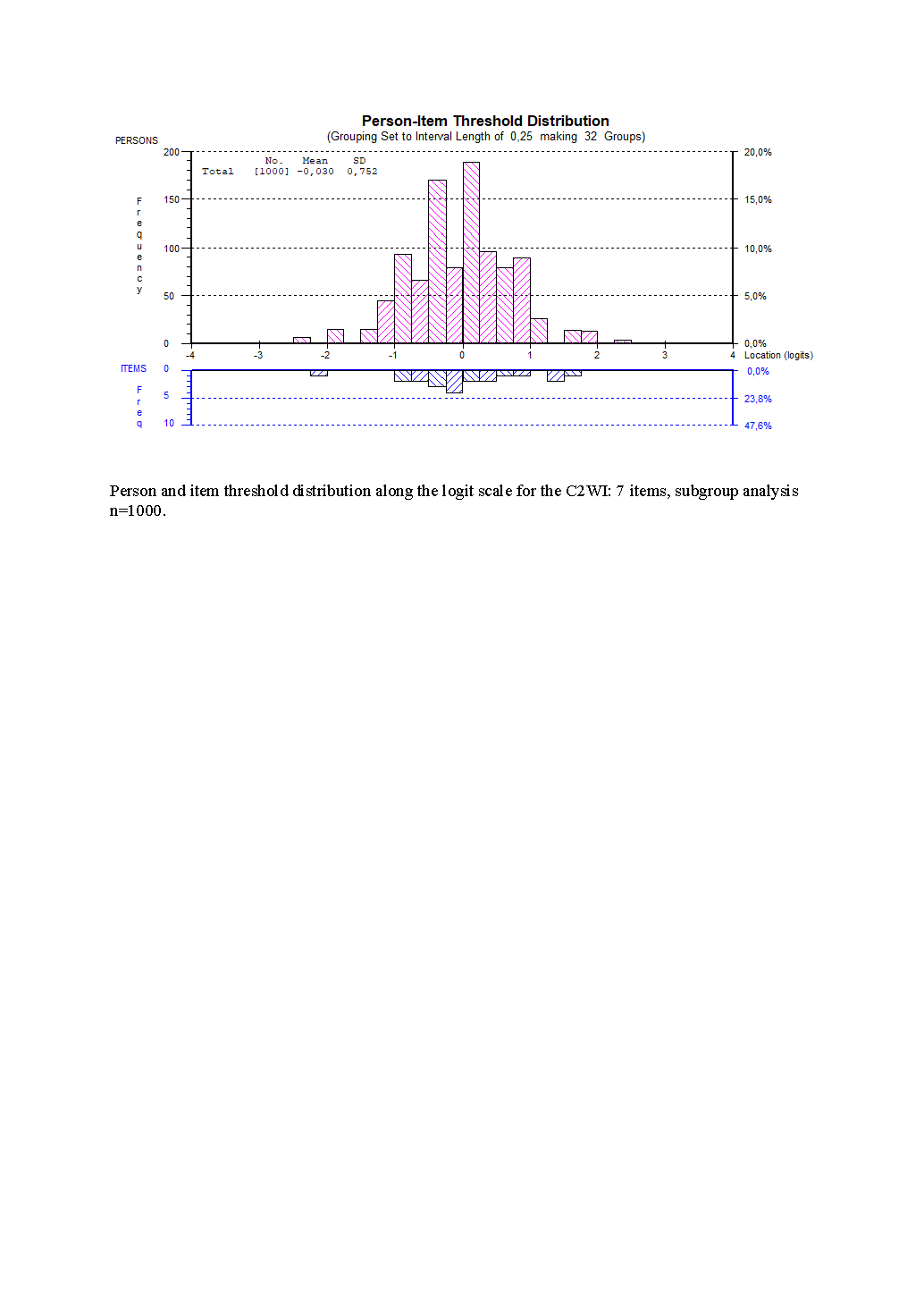

Supplement: ckaf001_Supplementary_Data [file ckaf001_supplementary_data.zip › ckaf001_Supplementary_Data/ejph-2024-06-om-0393-File015.tif]
